# Antiplasmodial, Antioxidant and Cytotoxicity Activity of Ethanol and Aqueous Extracts of *Khaya grandifoliola* Stem Bark

**DOI:** 10.1155/2023/8062453

**Published:** 2023-03-28

**Authors:** Gamago Nkadeu Guy-Armand, Yamssi Cedric, Noumedem Anangmo Christelle Nadia, Mounvera Abdel Azizi, Ngouyamsa Nsapkain Aboubakar Sidiki, Tientcheu Noutong Jemimah Sandra, Tako Djimefo Alex Kevin, Vincent Khan Payne

**Affiliations:** ^1^Department of Animal Biology, Faculty of Science, University of Dschang, P.O. Box 067, Dschang, Cameroon; ^2^Department of Biomedical Sciences, Faculty of Health Sciences, University of Bamenda, P.O. Box 39 Bambili, Cameroon; ^3^Department of Microbiology, Hematology and Immunology Faculty of Medicine and Pharmaceutical Sciences, University of Dschang, P.O. Box 96, Dschang, Cameroon; ^4^Department of Animal Organisms, Faculty of Science, University of Douala, P.O. Box 24157, Douala, Cameroon

## Abstract

**Background:**

Malaria is a serious public health problem, especially in sub-Saharan Africa. The aim of this study was to scientifically provide baseline information on the use of *Khaya grandifoliola* stem bark as an antimalaria drug by traditional healers.

**Method:**

The stem barks of *K.grandifoliola* were harvested and dried to obtain powder, and fifty grams of the powder were soaked in ethanol and hot distilled water respectively, for the preparation of ethanol and aqueous extracts, then dried in an oven at 40°C for the ethanol extract and 50°C for the aqueous extract. *Plasmodium falciparum* strains 3D7 sensitive and Dd2 resistant to chloroquine, were used to evaluate in *vitro* antiplasmodial activity using SYBR Green. The ability of the extracts to prevent oxidative stress was assessed by trapping 2, 2′-diphenyl-1-picrylhydrazyl (DPPH); nitric oxide, hydrogen peroxide and ferric reducing power. The cytotoxicity test of the extracts was carried out on RAW 264.7 cell lines and on erythrocytes. The data obtained were entered in the Excel software, then in Graph pad where the IC_50_ was calculated and the curves plotted.

**Results:**

The fifty percent inhibition (IC_50_) of the antiplasmodial activity of the chloroquine-resistant strain PfDd2 were 54.27 ± 2.41 *μ*g/mL and 31.19 ± 4.06 *μ*g/mL respectively, for the aqueous and ethanol extracts. As for the Chloroquino-sensitive Pf3D7, IC_50_ of 53.06 *μ*g/mL was obtained for the aqueous extract and 28.03 ± 1.90 *μ*g/mL for ethanol. The DPPH radical scavenging activity presented IC_50_ of 104 *μ*g/mL for the aqueous and 2.617 *μ*g/mL for the ethanol extract; for the Nitric oxide (NO) presented an IC_50_ of 301 ± 21 *μ*g/mL for the aqueous extract 140.7 ± 21 *μ*g/mL for the ethanol; for hydrogen peroxide the ethanol and aqueous presented IC_50_ of 845.1 ± 21 *μ*g/mL and 509.4 ± 21 *μ*g/mL respectively. The cytotoxicity on RAW 264.7 cells presented High CC_50_ in particular >1000 *μ*g/mL and 467.4 *μ*g/mL respectively for the aqueous and ethanol extract.

**Conclusion:**

Extracts of *Khaya grandifoliola* exhibited antiplasmodial activity. The ability to inhibit oxidative stress as well as lower cell toxicity on RAW 264.7 and erythrocytes, is a good indicator. However, *in vivo* tests remain important in order to confirm the use of this plant for the treatment of malaria.

## 1. Background

Malaria is the most important global parasitic endemic disease with approximately 3.3 billion people at risk in the world [1]. In 2020, with the COVID-19 pandemic, there are about 13 million new cases of illness worldwide and more specifically in Africa and around 69,000 new cases of death [2]. It is the primary medical concern of many countries in sub-Saharan Africa, where it is responsible for 95% of clinical cases and 96% of deaths, 80% of which are children under 5 years old [2].

In Cameroon, although there is an improvement in the malaria monitoring system, the National Malaria Control Program reports an increase in mortality (18.3%) and morbidity (28%) [3]. The shortage of antimalarial drugs and their relatively high cost partly explain this increase in malaria morbidity and mortality [3]. Added to this is the low efficacy of the antimalarial therapeutic arsenal of drugs and insecticides due to resistance recorded in Cameroon [4]and in some Asian countries [5]. To overcome these problems there is a pressing need to develop vaccines and new antimalarial drugs. More than 95% of drugs authorized on the market are of natural origin (Quinine and Artemisinin) or synthetic(Chloroquine) [6, 7]. In some communities in Africa, such as Guinea, Nigeria excess mortality due to malaria has been reduced thanks to the ability of traditional medicine to control the disease [8, 9].

According to Nadia et al. [10] and Abdel Azizi et al. [11] when a human host is infected with the Plasmodium parasite, these parasites stimulate an overproduction of free radicals to fight against infection. These free radicals are not only toxic to the parasite but equally toxic to the host. So it will be of paramount importance to have a drug that will possess antiplasmodial and antioxidant properties. In Cameroon, more than 200 plant species have been identified for their antiplasmodial properties. Traditional healers in Western Cameroon use the stem bark of *Khaya grandifoliola* for the treatment of malaria and salmonellosis. The work of Kodjio et al. [12] showed good anti-salmonellosis activity.

Previous work in Cameroon has demonstrated the anti-salmonellosis efficacy of various *Khaya grandifoliola* extracts [13, 14]. The aim of this study was to scientifically provide baseline information on the use of *Khaya grandifoliola* as an antimalaria drug in order to justify its usage by traditional healers in Cameroon.

## 2. Material and Methods

### 2.1. Collection and Identification of Plants

The leaves, flowers, fruits of *Khaya grandifoliola* (used for the identification of the plant) and stem barks (used for the preparation of the different extracts) were collected in June 2021 in the city of Foumbot, West Region of Cameroon and identified at the National Herbarium in Cameroon. A voucher specimen was deposited with the identification number 52658/HNC.

### 2.2. Preparation of Ethanol and Aqueous Extracts

Ethanol solvent was used for the extraction because during the survey traditional practitioner use fermented palm wine (ethanol) or infusion to prepare this remedy.

These extracts were prepared according to the method described by Wabo et al. [15]. The stem bark of *Khaya grandifoliola* was air-dried at room temperature under shade and pulverized using an electrical grinder under strict hygienic conditions. For the ethanol extract, 100 g of the powder was introduced into one litre of 95% ethanol and homogenised. The mixture was stirred daily for 72 h. The homogenate was filtered using cotton and whatman paper number 1. The filtrate obtained was dried in an oven at 40°C to obtain the ethanol extract. For the aqueous extract, distilled water was heated at 100°C and one litre was introduced into 100 g of powder and the mixture was allowed to cool and filter using cotton and whatman paper number 1. The filtrate was then dried at 45°C in order to obtain the dried aqueous extract.

### 2.3. *In vitro* Evaluation of Antiplasmodial Activity

#### 2.3.1. Plasmodium falciparum Cultured

*Plasmodium falciparum* strains were cultured using the Trager et Jensen Method [16] with slight modifications. Briefly, the multi-resistant PfDd2 and chloroquine-sensitive Pf3D7 strains were cultured in fresh human red blood cells of group O^+^ at 4% hematocrit in complete RPMI medium (Gibco, UK) supplemented with 25 mM HEPES (Gibco, UK), 0.50% Albumax I (Gibco, USA), 1X hypoxanthine (Gibco, USA) and 20 *μ*g/mL gentamicin (Gibco, China)] and incubated at 37°C in a humidified incubator consisting of 92% N_2_, 5% CO_2_ and 3% O_2_. This medium was replaced daily to facilitate growth of the parasites in the culture.

#### 2.3.2. Synchronization of Cultures

The parasite culture was synchronised at the same evolutionary stage (Trophozoite) by using 5% D. sorbitol, before evaluation of the antiplasmodial effect of *Khaya grandifoliola.*

#### 2.3.3. *In vitro* Antiplasmodial Activity Using SYBR Green

The *in vitro* antiplasmodial activity was evaluated based on the method described by Smilkstein et al. [17] with slight modification. Briefly, 10 *μ*L of various plant extract concentrations were introduced into a 96 cell microtiter plate. A parasite suspension of 90 *μ*L was added to each well to give a final extract concentration of 200–0.001280 *μ*g/mL. Artemisinin and chloroquine were used as positive control while 0.1% DMSO was used as the negative control. The plates were then incubated for 72 h in a CO_2_ incubator. After 72 h of incubation, 200 *μ*L of SYBR green were added and then incubated for 1 hour at 37°C in darkness. The fluorescence was measured at an excitation and emission wavelength of 485 and 538 nm respectively, using a Tecan Infinite M200 microplate reader. The IC_50_ for each extract was determined. The Resistance Index [RI] was calculated using the formula:

(1) $$\begin{array}{c} RI=\frac{IC50\;of\;PfDd2}{IC50\;of\;3D7}\text{.} \end{array}$$

### 2.4. *In vitro* Antioxidants Activity of Aqueous and Ethanol Extracts of *Khaya grandifoliola*

#### 2.4.1. Radical Scavenging Activity of 2,2 Diphenyl-1-Picrylhydrazyl (DPPH)

The DDPH radical scavenging activity was conducted according to the method described by Brand-Williams et al. [18] with slight modification. Briefly, the plant extracts and vitamin C were prepared in methanol to give a final concentration of 1; 3;10; 30; 100 et 300 *μ*g/mL and the absorbance was measured at 517 nm. DPPH was added and incubated for 20 minutes and the absorbance was read. The experiment was conducted in triplicate. The radical scavenging activity was calculated as follows:

(2) $$\begin{array}{c} RSA=\frac{Absorbance\;of\;control-Absorbance\;of\;sample}{Absorbance\;of\;control}x100\text{.} \end{array}$$

#### 2.4.2. Nitric Oxide Radical Scavenging Assay

The Nitric oxide radical scavenging activity was evaluated using the method described by Cheraff, [19]. The aqueous and ethanol extracts were dissolved in 3.53 mL of phosphate buffered saline (ph = 7.4). A volume of 1520 *μ*L of sodium nitroprusside was added respectively, into each sample containing 180 *μ*L of extracts and vitamin C (at the various final concentrations of 1, 3, 10, 30, 100, 300 *μ*g/mL). An equivalent volume of 1% sulfanilamide was added to 500 *μ*L of each sample and incubated for 5 min at room temperature in darkness. Naphthyl Ethylene Diamine (NED, 0.1%) was introduced into the mixture and incubated in darkness for another 5 minutes. The absorbance was read 30 minutes later with a spectrophotometer (BIOBASE-BK-D560) at a wavelength of 530 nm. The radical scavenging activity (RSA) expressed as a percentage was calculated as follows:

(3) $$\begin{array}{c} RSA=\frac{Absorbance\;of\;control-Absorbance\;of\;sample}{Absorbance\;of\;control}x100\text{.} \end{array}$$

#### 2.4.3. Scavenging Hydrogen Activity

The method of El-Haci et al. [20] was used to evaluate the peroxide scavenging activity. Four [21] mL of aqueous and ethanol extract were prepared in distilled water at different concentrations and mixed with 0.6 mL of a H _2_ O _2_ solution previously prepared with a buffer solution (0.1 M pH 7.4). The absorbance of the solution was measured at 230 nm after 10 min of incubation against a blank solution containing the extract without H_2_O_2_. The hydrogen peroxide scavenging activity was calculated as follows:

(4) $$\begin{array}{c} \%\;of\;H_{2}O_{2}\;RSA=\frac{Absorbance\;of\;control-Absorbance\;of\;sample}{Absorbance\;of\;control}x100\text{.} \end{array}$$

#### 2.4.4. Ferric Reducing Power

The Fe^3+^ to Fe^2+^ reduction test was carried out according to the method described by Pan et al. [22] with slight modifications. Two tubes containing 200 *μ*L of extract and vitamin C solution were added 0.5 mL of buffer solution (200 mM, pH 6.6) and 0.5 mL of potassium ferricyanide solution [K_3_Fe(CN)_6_] (30 mM). The tubes were incubated for 10 minutes at 37°C. The absorbance was measured at 700 nm using a spectrophotometer (BIOBASE-BK-D560).

### 2.5. Cytotoxicity Test

#### 2.5.1. Haemolysis Test

Healthy erythrocytes were used to perform the haemolysis test according to the method described by Sinha et al. [23]. Briefly, 500 *μ*L of a suspension of healthy erythrocytes of blood group O^+^ prepared at 4% hematocrit in incomplete RPMI1640 in the presence of 500 *μ*L of extract at different concentrations was incubated. The positive control and the negative control were prepared respectively, with Triton X-100 at 0.5% (for 100% haemolysis) and the erythrocyte suspension in the incomplete culture medium at 4% hematocrit. The final concentrations in the test plates varied from 1000 to 62.5 *µ*g/mL for the extracts and 0.5% for Triton X-100. The plates were incubated at 37°C for 3 hours in a CO_2_ incubator. After centrifugation at 2500 rpm/3 min, the absorbance of the supernatant was measured at 540 nm using the Infinite M200 microplate reader (Tecan). The degree of haemolysis of the various extracts was calculated as follows

(5) $$\begin{array}{c} Percentage\;of\;haemolysis\;\left(\%\right)=\frac{\left(OD\;extract-OD\;Negative\;control\right)}{OD\;Positive\;control}*100\text{.} \end{array}$$

#### 2.5.2. Cytotoxicity Test on RAW 264.7 Cells

This test was carried out according to the method described by Karakas et al. [24]. Seeding of macrophages was first done in 96-well cell microtiter plate at a density of 104 cells in 100 *μ*L of complete medium. The macrophages were incubated for 24 hours at 37°C, in a CO_2_ incubator. Each sample solution at various concentrations was added and incubated for 48 hours under the same experimental culture conditions. Cell proliferation was evaluated by adding 10 *μ*L of resazurin solution at a concentration 0.15 mg/mL to each well followed by 4 h of incubation under the same culture conditions. Fluorescence was then measured using a Tecan (Infinite) M200 microplate reader at an excitation/emission wavelength of 530/590 nm respectively. Results were expressed as 50% cytotoxic concentrations (CC_50_).

#### 2.5.3. Qualitative Phytochemical Screening

The extracts were tested for the presence of Sterols, alkaloids, Triterpenoids, saponins, Anthocyanins and anthraquinones using standard procedures described by Harbone [25].

#### 2.5.4. Total Phenolic and Flavonoid Contents

The Total Phenolic content was determined using the method described by Singleton and Rossi [26]. While the Flavonoid content was determined using method described by Djeussi et al. [27].

### 2.6. Statistical Analyses

The data were first entered into Microsoft Excel 16.0 Software to calculate the percentage of inhibition. Then imported into Graph pad Software Version 8.4 for the calculation of the IC_50_ from the concentration response curve obtained by plotting the logarithm of concentration as a function of the percentage of inhibition.

## 3. Results

### 3.1. *In vitro* Antiplasmodial Activity of *Khaya grandifoliola*

Table 1 presents the IC_50_ of the aqueous and ethanol extract of *Khaya grandifoliola* against *Plasmodium falciparum* sensitive 3D7 and the resistant Dd2 to Chloroquine (CQ). It follows from this Table that the aqueous extract had moderate activity, unlike the ethanol extract which is active against the 2 strains.

### 3.2. Antioxidant Activity of *Khaya grandifoliola*

#### 3.2.1. Radical Scavenging Activity of 1, 1-Diphenyl-2-picrylhydrazyl (DPPH)

**Figure 1:**
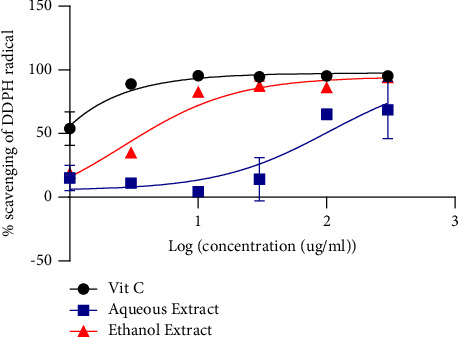
DPPH scavenging activity.

Figure 1 shows the scavenging activity of DPPH. The ethanol extract (2.61 *μ*g/mL) presented good DPPH radical scavenging activity compared to the aqueous extract (104 *μ*g/mL).

#### 3.2.2. Nitric Oxide Scavenging Activity

**Figure 2:**
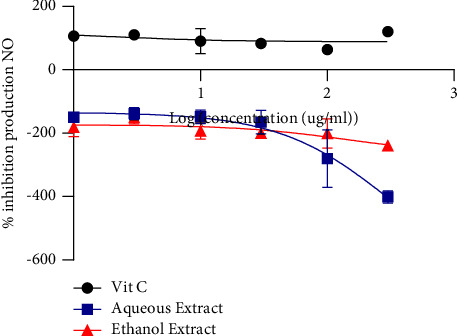
Inhibition of NO by the aqueous and ethanol extracts of *Khaya grandifoliola.*

Figure 2 shows the curves expressing inhibition of the NO radical by the aqueous and ethanol extracts of *Khaya grandifoliola*. It appears that the aqueous had an IC_50_ of 301 *μ*g/mL and ethanol 140.7 *μ*g/mL. These values indicate that aqueous and ethanol extracts do not have NO scavenging activity compared to the Ascorbic acid which presented good NO scavenging activity.

#### 3.2.3. Hydrogen Peroxide (H_2_O_2_) Scavenging Activity

**Figure 3:**
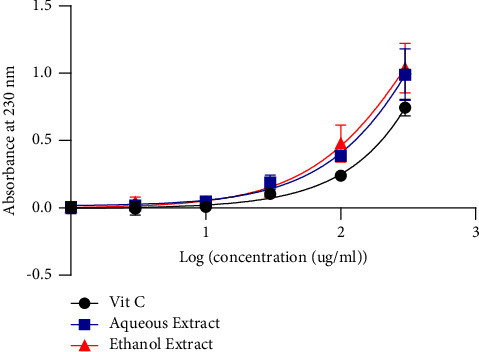
Hydrogen peroxide scavenging activity.

Figure 3 shows hydrogen peroxide (H_2_O_2_) scavenging activity. It appears that the aqueous and ethanol extract presented IC_50_ of 845.1 *µ*g/mL and 509.4 *µ*g/mL respectively. These extracts had scavenging activity which was greater than Ascorbic acid (∼9291 µg/mL).

#### 3.2.4. Ferric Reducing Power

**Figure 4:**
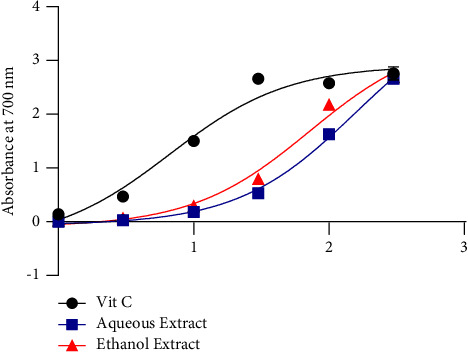
Ferric reducing power of *Khaya grandifoliola*.

Figure 4 expresses the ability of the aqueous and ethanol extract of *Khaya grandifoliola* to reduce iron. It appears that the aqueous extract (156.2 *µ*g/mL) and ethanol (71.77 *µ*g/mL) have a reducing power of iron, which is much lower than that of Ascorbic acid (6.672 *µ*g/mL).

### 3.3. Cytotoxicity Test

#### 3.3.1. Red Blood Cell Cytotoxicity Test (Haemolysis Test)

**Figure 5:**
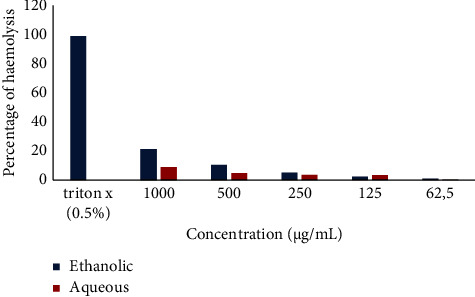
Haemolytic effect of the aqueous and ethanol extract of *Khaya grandifoliola.*

Figure 5 shows the effect of the aqueous and ethanol extract of *Khaya grandifoliola* on human erythrocytes. It follows from the analysis of this Figure that for all doses, the aqueous extract showed lower haemolytic activity than that of the ethanol extract. The highest haemolysis percentage was observed for the ethanol extract at a dose of 1000 (*μ*g/mL).

#### 3.3.2. Cytotoxicity on RAW 264.7 Cells

Table 2 shows the effects of aqueous and ethanol extracts on RAW 264. 7 cells. It appears that the CC_50_ of the aqueous and ethanol extract were >1000 *μ*g/mL and 467.4 *μ*g/mL respectively.

#### 3.3.3. Qualitative Phytochemical Screening

Table 3 shows the phytochemical screening of the aqueous and ethanol extracts of Khaya grandifoliola sterm bark. It follows from the analysis of this table that the aqueous extract contains alkaloids, saponins, triterpenoids, anthocyanins, and anthraquinone. Similarly, the ethanolic extract contains the same compounds except saponins.

#### 3.3.4. Total Phenolic and Flavonoid Contents

**Figure 6:**
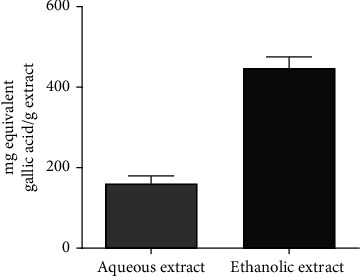
Total flavonoids content of the aqueous and ethanol extracts.

**Figure 7:**
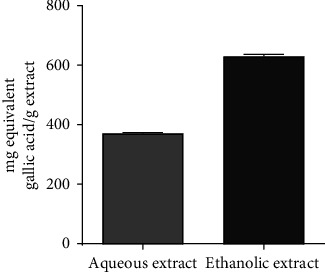
Total phenolic content of the aqueous and ethanol extracts.

Figure 6 shows the amount of flavonoid present in each extract. It appears from this figure that the ethanol extract (448.9 ± 68.85 mg/g) content more flavonoids than the aqueous extract (162.2 ± 48.20 mg/g).  Figure 7 shows the total phenolic content of the aqueous and ethanol extracts. Similarly, more phenolic compounds where found in the ethanol extract (372.4 ± 7.328 mg/g) compared to the aqueous extract (631.9 ± 16.44 mg/g).

## 4. Discussion

Antiplasmodial activity on the PfDd2 chloroquine-resistant strain was 54.27 ± 2.41 *μ*g/mL and 31.19 ± 4.06 *μ*g/mL respectively for the aqueous and ethanol extracts. As for the Chloroquino-sensitive Pf3D7 an IC_50_ of 53.06 *μ*g/mL for the aqueous extract and 28.03 ± 1.90 *μ*g/mL for ethanol were obtained. According to the classification criteria established by Kumari et al. [28] which states that a plant extract with IC50 < 5 *μ*g/mL is declared very active; between 5 and 50 *μ*g/mL is declared active; 50–100 *μ*g/mL moderate and >100 inactive. These results show that the ethanol extract was active while the aqueous extract had moderate activity both for 3D7 sensitive and Dd2 resistant to strain. Similar results were observed by Abdel Azizi et al. [11] when evaluating the *In Vitro* Antiplasmodial Cytotoxicity and Antioxidant Activities of *Lophira lanceolata* (Ochnaceae): A Cameroonian Plant Commonly Used to Treat Malaria. This could be explained by the nature of the solvent used for the extraction. Furthermore, the ethanol extracts content more phytochemical constituent compared to the aqueous extract as demonstrated in our results. According Koagne et al. [29] flavonoids could lead to more useful derivatives for the development of an antiplasmodial agent. The work of Azebaze et al. [30] demonstrated the antiplasmodial activity of isolated phenolic compounds. The stem bark of *Khaya grandifoliola* is rich in compounds such as Alkaloids, Flavonoid, Phenol, Terpenoids, Anthocyanins and Anthraqui-none [31]. One of these compounds such as phenols or flavonoid have antiplasmodial properties [32]. The high capacity of ethanol to extract these phytochemical constituents [33] could explain the better activity obtained with the ethanol extract. A similar study carried out in Nigeria on the Chloroquino-resistant W2 clone of *Plasmodium falciparum* showed an active activity of the aqueous extract of *Khaya grandifoliola* with an IC_50_ of 15.2 *μ*g/mL [34]. This difference observed between the antiplasmodial activity of the two aqueous extracts could be linked to the difference in the Plasmodium strains on which the extracts were evaluated, and also to the chemical composition of the plant which may vary according to certain characteristics such as climatic and edaphic factors [35].

The aqueous and ethanol extracts displayed less free radical scavenging ability than ascorbic acid in the DPPH and NO scavenging tests. Similar results on the Meliaceae plant *Entandrophragma cylindricum* revealed lower free radical scavenging ability of the methanol extract than Ascorbic acid [36]. Our findings, however, do not support those of Kodjio et al. [12] who demonstrated a more effective DPPH scavenging activity than Ascorbic acid. The low concentration of phenolic compounds and flavonoids that can release H+ to assist the scavenging of DPPH may account for this discrepancy.

As for the hydrogen peroxide scavenging test, the aqueous extract (IC_50_ : 845.1 and ethanol (IC_50_ : 509.4) presented high scavenging activity compared to that of ascorbic acid (∼9291). This means that these extracts may have a greater power of hydrogen peroxide inhibition. This is an advantage for the host organism because although hydrogen peroxide, plays a role in the body's defense, it is often toxic [37].

The aqueous and ethanol extract presented a ferric reducing power lower than that of ascorbic acid. This result indicates that these extracts promote the production of Fe^2+^. The ethanol extract reduces the synthesis of Fe^2+^ more than the aqueous extract. Our results corroborate those obtained by Kodjio et al. [12] where the ferric reducing power of the ethanol extract was greater than the aqueous extract on the evaluation of the antioxidant activity of the stem bark of *K. grandifoliola*. This high antioxidant power could be due to the strong presence of flavonoids and phenolic compounds [11, 12, 38].

The cytotoxicity test on the erythrocytes revealed a negligible lysis of the erythrocytes at all concentrations. Similarly, a study carried out on aqueous leaf extracts of *Aerva lanata, Calotropis gigantea*, and *Elaeocarpus ganitrus* alone and in combination showed low cytotoxicity [39]. On the other hand, the work carried out on *Allium stracheyi Baker* showed strong erythrocyte lysis [40]. These differences could be justified by the low capacity of the aqueous and ethanol extract of *Khaya grandifoliola* to reduce iron.

These cytotoxicity tests on RWAN264.7 had CC_50_ of >1000 *μ*g/mL for the aqueous and 467.4 for the ethanol extract. According to the American National Cancer Institute, any extract considered cytotoxic has a CC_50_ < 30 *μ*g/mL [41]. These extracts are therefore considered non-toxic. Similar research by EL Souda et al. [42] showed that the volatile extract of *Khaya senegalensis* had moderate cytotoxicity on MCF7 cells with a IC_50_ of 79.7 *μ*g/mL. On the other hand, the same study found that *Khaya grandifoliola's* volatile extract showed a high level of cytotoxicity against MCF7 cells, with an IC_50_ of 21.8 *μ*g/mL. [42]. This would be supported by the high concentration of sesquiterpenes, particularly isocaryophyllene and humulene (caryophyllene), in the volatile extract of *K. grandifoliola*, which could be influenced by both the plant's collection site(region) and the parts of the plant used to prepare the extract.

## 5. Conclusion

The ethanol and aqueous extract of *Khaya grandifoliola* exhibited antiplasmodial activity against *Plasmodium falciparum* 3D7 sensitive and Dd2 resistant to chloroquine. These extracts presented good scavenging against most of the free radicals, and were non cytotoxic. This reflects a possible use of these extracts to avoid oxidative stress related to Plasmodium infection in the treatment of malaria. However, further *in vitro* studies are required to ascertain their antiplasmodial activities.

## Figures and Tables

**Table 1:** *In vitro* antiplasmodial activity.

Plant extract	IC50 ± SD (*µ*g/mL)		RI	Observation
	Pf3D7	PfDd2		
Aqueous	54.27 ± 2.41	53.06 ± 0.00	1.02	Moderate active
Ethanolic	31.19 ± 4.06	28.03 ± 1.90	1.11	Active

*Positive Control*				
Artemisinin (*µ*M)	0.033 ± 0.00024	0.04 ± 0.00067	0.94	N A
Chloroquine(*µ*M)	0.53 ± 0.0405	0.30 ± 0.00669	1.75	N A

IC_50_ inhibitory concentration 50; RI: resistance index, NA: not applicable.

**Table 2:** Effect of aqueous and ethanol extract of *Khaya grandifoliola* on RAW 264.7 cells.

Extract	IC_50_		CC_50_ (*μ*g/mL)	SI	
	Pf3D7	PfDd2		Pf3D7	PfQd2
Aqueous	53.065	54.275	>1000	>35.66	>19.47
Ethanol	28.035	31.195	467.4	16.672	19.065

**Table 3:** Phytochemical screening of *Khaya grandifoliola* extracts.

Extracts	Alkaloids	Sterols	Saponins	Triterpenoids	Anthocyanins	Anthraquinons	Flavonoids	Polyphenol
Aqueous	+	−	+	+	+	+	+	+
Ethanolic	+	−	−	+	+	+	+	+

+: present −: absent.

## Data Availability

All data generated and analysed are included in this research Article.
